# Development and evaluation of decision aids for people considering taking part in a clinical trial: a conceptual framework

**DOI:** 10.1186/s13063-019-3489-y

**Published:** 2019-07-05

**Authors:** Katie Gillies, Marion K. Campbell

**Affiliations:** Health Services Research Unit, University of Aberdeen, Health Sciences Building, Foresterhill, Aberdeen, AB25 2ZD UK

**Keywords:** Informed consent, Clinical trials, Decision aids, Decision support, Complex interventions, Conceptual framework

## Abstract

Ethical requirements of informed consent stipulate that patients approached to participate in a clinical trial be provided with written information that must cover key aspects of the trial. For consent to be deemed “informed”, potential participants should be provided with a range of information about the trials (e.g., the trial aims, the anticipated benefits and potential risks of the trial, and their right to withdraw consent at any time). However, it is well documented that simple provision of this information does not ensure that participants make truly informed decisions. Decision aids, tools that have been shown in a treatment and screening context to support better-quality decisions, are emerging as a possible vehicle to support decision making about trial participation. However, information on how they should best be developed and evaluated in a clinical trial context is lacking. Therefore, this article, drawing on theoretical and empirical insights, outlines a framework for the development and evaluation of decision aids for people considering taking part in a clinical trial.

## Background

The requirement for informed consent to be sought from potential participants when they are deciding whether to enter a clinical trial is enshrined in the Declaration of Helsinki and several other international and national regulations [1–3]. These regulatory requirements were established as a mechanism to protect participants from any undue harm from research [1]. The regulations stipulate that, for consent to be deemed “informed”, potential participants should be provided with a wide range of information elements, including the trial aims, methods, sources of funding, any possible conflicts of interest, institutional affiliations of the researcher, the anticipated benefits and potential risks of the trial, what happens at the end of the trial, and their right to withdraw consent at any time without reprisal [1–3]. During the informed consent process, potential participants are usually provided with a written patient information leaflet (PIL) summarising this information [3]. However, simply providing this information does not, of itself, ensure that consent is informed. Evidence from several studies illustrates that current approaches to informed consent for clinical trials—in particular, simple provision of information in the PIL—may be suboptimal [4–6]. For example, some have developed information leaflets without asking potential participants what information they consider key for supporting a decision to take part in a trial. Others have focused solely on the printed information without considering the conversation in which it is to be placed [5, 6]. Many of these deficits have been targeted for improvement through the development of interventions to improve information provision [4–7]. However, these interventions have tended to be narrowly focused on the content and structure of information provision and evaluate effectiveness by measuring outcomes such as understanding, knowledge and trial recruitment rather than potential wider outcomes such as decisional conflict or regret [4–7].

Evidence from the treatment and screening decision-making literature has identified that, for making “good” decisions, the provision of information alone is not sufficient [8]. “Good” decisions can be thought of as those that improve the match between the chosen decision and the features that matter most to the informed patient [8]. Providing ways for people to be able to compare options, facilitating the ability to make trade-offs between options, and allowing people to weigh up potential outcomes of decisions and assess how different outcomes matter to them have all been shown to promote “good” decisions [8]. These items, and others, are often included in so-called “decision aids”, formal tools which help people to participate in treatment (or screening) decisions that involve weighing up associated benefits and harms in the face of clinical uncertainty [8, 9]. Decision aids have been shown to positively influence various aspects of decision making across a range of treatment and screening decisions [8, 9].

Decision aids for people considering taking part in a clinical trial are emerging in the literature but remain sparse [10]. A handful of feasibility or full trials evaluating decision aids to inform decisions about trial participation are reported in the literature and others are in the planning stage [11–17]. Early randomised comparisons show that, in this setting, decision aids have potential promise with regard to improving key decision outcomes (knowledge, values clarification, and decision conflict) and not negatively affecting recruitment or intention to participate [14–16]. However, most examples of decision aids for trial participation are currently set within an oncology context; there is a paucity of examples in other disease areas and across a range of intervention choices. The reason for this current focus in oncology is unclear, although intuitively it may reflect a natural extension of the rich history of the use of decision aids for treatment decisions in this context. However, it is likely that different clinical contexts will generate specific (both clinically and when considering interventions being compared) issues for the design of decision aids and these will have to be accommodated for in the development process. As the appetite for decision aids for trial participation grows, it is important that these be developed in a rigorous and evidence-based way. There are several reports that outline the overall and stepwise process for developing decision aids for treatment and screening decisions [18, 19]. However, there is no counterpart that describes a comprehensive approach to the development of trial participation decision aids. To this end, this article describes a potential framework for the development and formulation of prototype decision aids to aid decisions of whether to participate (or not) in a clinical trial on the basis of theoretical and empirical insights. It describes the need to select an underpinning theoretical approach to inform the development process and steps that we believe should be undertaken to successfully develop and evaluate candidate decision aids in this context.

Within the general framework, the following steps are proposed:

- Selecting an underpinning theoretical approach to the development process
- Developing the decision aid
- Assessing and testing feasibility
- Evaluating the decision aid
- Implementing the decision aid in practice.

We illustrate the process by using case studies of the development and evaluation of decision aids for two exemplar trials. The first is a drug trial comparing two active drugs versus placebo for the treatment of ureteric stones (SUSPEND ISRCTN: 69423238, date of registration: 18 November 2010) and the other is a trial comparing two surgical procedures for the treatment of haemorrhoids (eTHoS ISRCTN: 80061723, date of registration: 8 March 2010). Further information on each of the host trials can be found in Table 1.

**Table 1:** Characteristics of host trials

Trial characteristics	eTHoS	SUSPEND
Clinical condition	Haemorrhoids	Ureteric stones
Trial design	Simple parallel design	Simple parallel design
Sample size, number	800	1200
Recruitment rate, percentage	74	56
Arms	2	3
Intervention	Surgery	Drug
	1. Traditional excisional haemorrhoidectomy	1. Calcium channel blocker
	2. Stapled haemorrhoidopexy	2. Alpha blocker
		3. Placebo
Blinding	Participants and outcome assessors (for patient-reported outcomes)	Participants, caregivers and outcome assessors
Number of sites	31	24
Primary outcome (clinical or patient-reported and timing)	Patient-reported at 2 years post-randomisation	Clinical at 4 weeks and patient-reported at 12 weeks post-randomisation
Host trial participant characteristics		
Age in years, median (interquartile range)	49 (20–40)	44 (34–52)
Sex (percentage who are females)	48	19

### Selecting an underpinning theoretical approach to the development process

Guidance previously written to inform the development of decision aids for treatment decisions endorses the identification or development of a guiding theoretical framework to support decision-aid development [18–20]. Several theories and conceptual frameworks have been used to develop treatment and screening decision aids [21]. We reviewed a range of possible candidate frameworks and identified the Ottawa Decision Support Framework (ODSF) as being a highly relevant theoretical framework to use for development of trial participation decision aids. The ODSF is a descriptive framework that is based largely on the construct of decisional conflict (shown to be salient in a trial participation context [22]) but also includes input from various theories in psychology, social psychology, social support, and economics [22]. The ODSF assumes that there are unmet decisional needs (e.g., knowledge deficits, decisional conflict, unrealistic expectations, unclear values, and unmet support) that, once addressed, will result in improvements in decision quality. This maps very well onto the known issues within trial participation decisions as they are known to be preference-sensitive decisions—decisions largely influenced by a patient’s preferences and values [23].

We also recognised that decision aids involve multiple components, interacting systems and processes; as such, they also naturally fit the definition of a “complex intervention” [11, 24]. Therefore, when considering how to develop our trial participation decision aid, we also deemed it important to appeal to the international Medical Research Council (MRC) guidance on developing and evaluating complex interventions [25]. The MRC guidance suggests an iterative model of development and testing of complex interventions through defined stages: development, feasibility, evaluation and implementation. An integration of both the MRC guidance and the OSDF principles thus formed the underpinning theoretical approach to our development work.

### Developing the decision aid

- *Identifying the need for trial participation decision aids*

The guidance on the development of treatment and screening decision aids recommends that the first step focus on determining a need for the requirement of a decision aid (i.e., defining the scope and addressing decisional needs) [18–20]. Our justification for the development of a decision aid is based on two elements: (a) in the context of trial participation, regulatory requirements stipulate that the provision of information is a mandatory pre-requisite to informed consent, and (b) there is no evidence on the optimal method to support people’s decision making about trial participation (see above). The guidance on developing complex interventions and decision aid development guidance complement the requirement to identify the “need” for the decision aid by recommending that any complex intervention being developed incorporate what is already known on the interventions of interest [19, 25].

We also searched for evidence to ensure that an appropriate decision aid did not already exist. In our search for evidence, we identified (a) reviews of decision aids for treatment and screening and (b) reviews of interventions (not solely decision aids) to improve informed consent to trials [4, 7, 8]. However, we were not able to identify any existing systematic review of decision aids specifically for people considering trial participation. Therefore, we conducted a wider search to review all of the available evidence in this area [10]. The results of this wider review also supported the conclusion that there remained a gap in the evidence base for the development of a decision aid in this field (only one eligible study was identified but it did show evidence of benefit) in this context, but the review also informed the shape and scope of the decision aid (see below).

In addition to conducting a review of the published evidence, we also contacted relevant experts to identify any potentially relevant new, ongoing or unreported research in this area. Specifically, we conducted a formal survey of the Directors of the UK Clinical Research Collaborations (UKCRC) registered Clinical Trial Units [26]. We also canvassed the shared decision-making community through the use of social media platforms. The survey (which did not identify any ongoing studies in the field at the time) and the systematic review (which identified one study suggestive of benefit) provided evidence to support the need for further research in this area. Thus, on the basis of both the theoretical and empirical evidence, we concluded there was a continuing need for the development of a decision aid for trial participation.

b. *Defining the scope*

The literature review conducted above showed that considerations for the development of a decision aid would likely differ depending on factors such as the phase of trial, whether prospective or retrospective consent (e.g., for emergency trials) was being sought, and whether proxy decision makers were those who would provide the consent. As such, it was deemed crucial to explicitly outline the scope of the decision aid at the outset of the development phase. In our context, the scope and context for our prototype decision aids were phase III effectiveness trials of interventions in adults with the capacity to give their autonomous prospective consent to participate (or not) in a clinical trial.

c. *Identifying the content requirements*

**Fig. 1:**
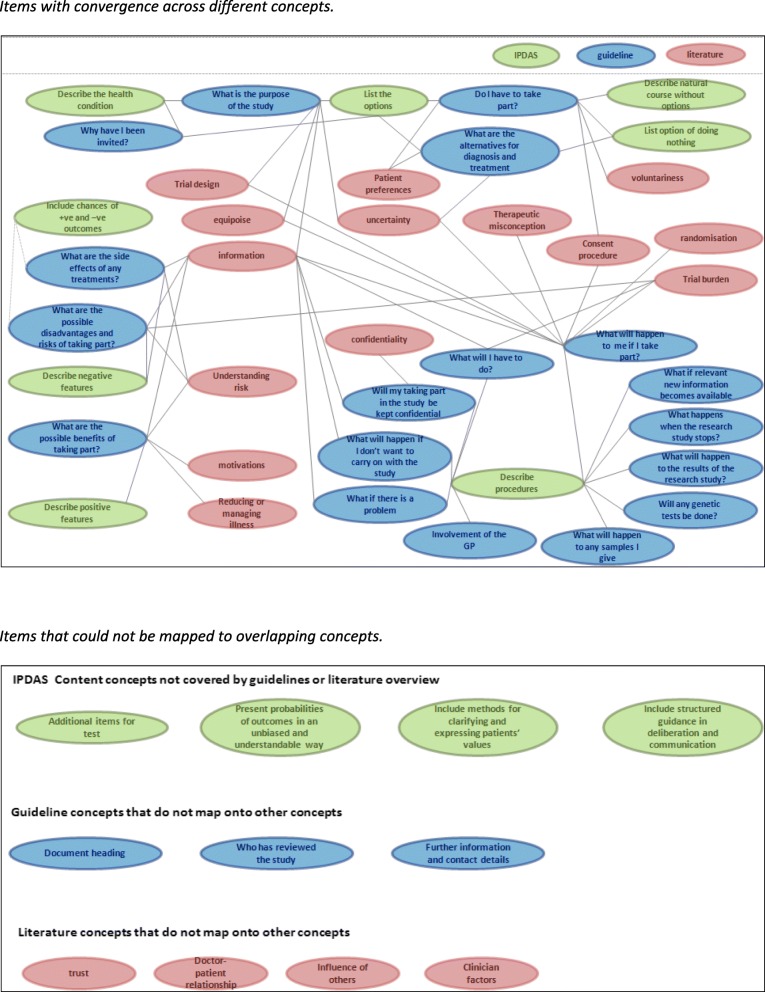
Content mapping of International Patient Decision Aid Standards (IPDAS)/literature on informed consent for trials/informed consent guidelines. *Items with convergence across different concepts. Items that could not be mapped to overlapping concepts*

To determine what the content requirements of any trial participation decision aid would be, we first conducted a concept-mapping exercise (a process that depicts relationships between individual concepts). This concept-mapping process brought together the evidence we identified from the literature relating to factors that influence an individual’s decision about whether (or not) to participate in a clinical trial, the regulatory guidance on what information participants have to and should be told when considering trial participation, and the International Patient Decision Aid Standards (IPDAS) content items [27]. This information was broken down into individual items and “mapped” to identify areas of convergence and divergence across the three sources (Fig. 1). Items were assessed for duplication of concepts and were reduced accordingly to generate a list, supplemented with the divergent items, to produce a final candidate item set.

This item set formed the basis of a Delphi survey to assess consensus across stakeholders as to the core set of items that should be included in a final decision aid. The Delphi survey asked stakeholders (trialists, research nurses, ethics committee chairs, decision support experts and patients—both trial-naïve and trial-experienced) to rate the importance of each item for inclusion in a trial participation decision aid—in essence to identify the core information required to support decision making in participant information for clinical trials. The full results of this Delphi survey have been published previously [27]. In summary, the Delphi survey results showed that many of the items deemed important for decision making in treatment and screening (e.g., information to help patients determine what matters most to them, ensuring that the information is balanced, and guidance on how to make a decision) are also considered important in this context [27].

In addition to conducting the Delphi survey, we conducted a review of existing PILs for clinical trials by using a tool based on the IPDAS. The purpose of conducting this review was to determine whether and how existing PILs fulfil the requirements of decision aids. Again, the results of this work have been published elsewhere [28]. In brief, this work identified key areas where existing PILs were lacking; as such, these areas (such as presenting probabilities and clarifying and expressing values) should be included in future trial participation decision aids [28].

The items agreed to by at least one stakeholder group in the Delphi survey as being important (after two rounds of rating) and those key informational gaps identified from the review of PILs (total of 60 items across both pieces) were carried through to the prototype development stage, described in more detail below. Table 2 provides examples of the types of informational items identified during this stage.

d. *Creating the prototype decision aid*

**Table 2:** Example information items identified from Delphi survey and patient information leaflet review and included in prototype decision-aid development

	Section	Item
A	The decision support tool development process	Finding out what information potential participants need to prepare them to discuss trial participation
		The decision support tool was tested out with recruiters who are actively engaged in discussing trials with potential participants.
B	Providing information about trial participation and standard care	The decision (i.e., trial participation or not) that needs to be considered is adequately described.
		The decision support tool presents information about the advantages/benefits of trial participation.
		The decision support tool presents information about the advantages/benefits of non-participation.
		The decision support tool explains that taking part in the trial is voluntary.
C	Presenting information on the likelihood (i.e., chance) of receiving different treatments	The decision support tool presents textual information (i.e., information in words) on the chances of receiving specific treatments. For example, for a trial of surgery versus medical treatment, you have a 1 in 2 chance of getting surgery if you take part in the trial or 100% chance of getting medical treatment if you do not take part in the trial.
		The decision support tool provides more than one way of explaining the chances (e.g., words, numbers and diagrams).
		The decision support tool presents information about advantages and disadvantages of trial participation that includes the likelihood that they will happen.
D	Determining what matters to participants	The decision support tool describes the features of trial participation and standard care to help participants imagine what it is like to experience these options. For example, “Surgery A may result in pain in your right knee. People who experience this pain may find it hard to move around following surgery”.
		The decision support tool asks participants to think about which advantages and disadvantages of trial participation and standard care matter most to them.
E	Using stories from other participants	The decision support tool provides stories of other participants’ experiences of deciding to participate (or not) in a trial.
		The decision support tool provides stories that represent a range of experiences (positive and negative) of taking part (or not) in a trial.
F	Decision guidance	The decision support tool provides a step-by-step way to make a decision about trial participation (e.g., by using a list or worksheet that outlines the steps or by developing the decision support tool in such a way that it guides the participant through the decision).
G	Disclosing conflicts of interest	The decision support tool reports who is organising and funding the research.
		The decision support tool contains details of who has reviewed (from both a scientific and ethical perspective) the trial.
H	Balancing the presentation of options	The advantages and disadvantages of trial options and standard care are presented with equivalent detail (e.g., using similar fonts, order, and display of statistical information).
I	Using plain language	The information is written at a level that can be understood by at least half of the participants for whom it is intended.
		The information provides ways other than reading (e.g., audio, video, or in-person discussion) to help participants understand information.
J	Basing included information on up-to-date scientific information	The decision support tool describes the quality of the scientific evidence (e.g., quality of research studies).
		The decision support tool uses evidence taken from studies on participants that are similar to the participants who would use the information (e.g., age and gender).

**Fig. 2:**
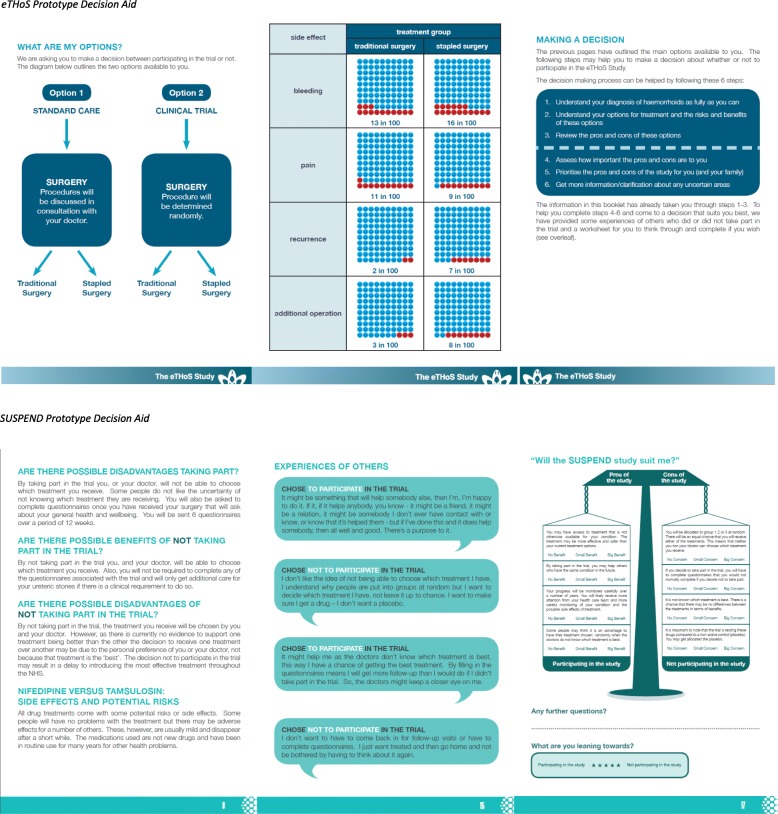
Sample pages from prototype decision aids to support trial participation decisions. *eTHoS Prototype Decision Aid SUSPEND Prototype Decision Aid*

To create the actual prototype decision aid, we worked through the key elements of the ODSF (introducing the decision, describing potential benefits and risks and probabilities in varying formats, clarifying and communicating values, and assessing unresolved needs and next steps). We used the informational items generated from the Delphi survey and the PIL review to populate the appropriate sections. Presentation of information was developed through working with graphic designers. Sample pages from the decision aids for each of the two exemplar trials are presented in Fig. 2.

### Assessing and testing feasibility of the prototype decision aids

The ODSF recommends that during the decision aid development and piloting process there be stakeholder engagement and that preferences for intervention delivery be incorporated [22]. To undertake feasibility and piloting of the new prototype decision aids, we undertook an exploratory pilot of the prototype decision aids (so-called “alpha testing” [19]). During the exploratory pilot, we also sought stakeholders’ opinions about what the main objective of the decision aid should be. Once the main objective is explicit, appropriate outcomes to measure whether that objective had been met (and thus whether the decision aid had been effective) can be identified. Many of the responders suggested that support for informed decision making (or similar e.g. informed choice) be the most important outcome to determine the effectiveness of a decision aid. However, others considered trial relevant outcomes such as recruitment and retention rates to be the main focus for evaluation (unpublished data).

It has also been noted that it is important for developers to consider how the choice of delivery mode will lend itself to review by ethics committees (i.e., if a web-based platform is used, printouts of the content may need to be provided for review) [29]. To date, most PILs have been paper-based and hence we elected to deliver the decision aids we developed as printed A5 booklets. Not surprisingly, the stakeholders in our pilot had varying preferences (both within and between groups) relating to mode of delivery of the trial decision aids. Benefits cited for the paper format included the ability to take it away to discuss with others [11]. However, others would have preferred it in electronic format, and several noted the importance of context with regard to the trial population being recruited when considering options about delivery [11]. Mode of delivery should be considered during development with the potential to adapt delivery method according to local parameters or specific patient preferences. The results of this exploratory pilot have been published elsewhere [11].

#### Stakeholder input

Involvement of multiple stakeholders and iterative rounds of empirical, formative research are essential in developing an intervention that is both acceptable and implemented by all end users. In the case of trial participation decision aids, we believe that it is important to consider the perspectives of all those who have a stake in the intervention, from designers (trial managers, clinicians and methodologists) to reviewers (ethics committee and sponsor) and consumers (potential trial participants and deliverers such as research nurses) [26]. In our pilot assessment of acceptability, we included a range of stakeholders, specifically research nurses, trial managers, ethics committee chairs, clinical investigators, and potential trial participants [24]. This process of engagement with intervention “users” (not just during feasibility but throughout the process of development) has similarities with user-centred design [30]. User-centred design is an iterative method for optimising “user” experience (and, indirectly, effectiveness) of a service, system or product [30]. This framework is being adopted by researchers as a way to review user involvement (and develop best practice guidance) during the development of treatment decision aids [31].

#### Assessment of acceptability

It is further recommended by the ODSF that the chosen method of assessment (i.e., large-scale investigation or small-scale in-depth exploration) should be appropriate to answer the specific concerns the research team have about acceptability [22]. Our study used semi-structured qualitative interviews to explore stakeholders’ perspectives about the acceptability and (potential) usefulness of the prototype decision-aid interventions [24]. The benefits of this approach were that it allowed an in-depth assessment of individuals’ perspectives on the pros and cons of trial participation decision aids and allowed clarification of any misunderstandings in relation to the intervention. However, this did mean that the included sample was small and this in itself may present limitations (i.e., were the perspectives of those in our sample different from others in the population). Some studies investigating the usefulness of treatment decision aids have used quantitative surveys to assess intervention acceptability [32]. It may also be that, through rounds of testing and user feedback, small exploratory studies assessing acceptability could feed into larger-scale survey work. This survey work may have the potential to facilitate the implementation phase through engaging with end users early on.

### Evaluation

The final phase of any decision-aid development process is the formal evaluation.

- *Choice of evaluation design*

The most robust method to evaluate the large-scale effectiveness of any healthcare intervention is acknowledged to be the randomised controlled trial (RCT) as it minimises selection bias and allows reliable estimates of effect to be concluded [33]. In the context of evaluating a decision aid, this would require embedding a formal RCT of the effectiveness of decision aid versus standard trial information on the consent process within a host clinical trial.

It is also important to consider what type of randomised design (e.g., an individually randomised design and a cluster trial design) is most appropriate to assess the effectiveness of these interventions. Of the randomised comparisons of trial participation decision aids (defined using the IPDAS [34]) conducted to date, all have been conducted as individually randomised two-arm parallel trials [14–16]. However, the use of a stepped-wedge or cluster design, where groups/clusters (e.g., sites) are randomised to use the decision aid (or the traditional consent process), may be more appropriate as it would minimise any contamination effect that might be introduced through the training of staff in the use of the decision aid. One such study is ongoing: a stepped-wedge RCT to assess effectiveness of a decision aid to reduce decisional conflict in breast cancer patients considering participation in a prospective cohort study [17].

Given that a decision aid is a complex intervention with interacting components (the decision-aid process involves interactions between a participant, the aid and the person delivering the aid), it is also important for the evaluation to be designed to ensure that any assessment of effectiveness can be directly attributed to the decision-aid process itself (and not to any wider system influences such as provision of supplementary leaflets or information via the person delivering the aid). A concurrent process evaluation (see below) will aid the formal evaluation in this regard.

b. *Choice of primary outcome*

Another key consideration in the design of the evaluation is the choice of primary outcome and any related secondary outcomes and the relevance of these for the decision-aid trial and the host trial (i.e., impacts on recruitment and retention). All of the previous studies have chosen decisional conflict or knowledge (or both) as their primary outcome [14–17]. Whether or not these are the most appropriate primary outcomes to use for the evaluation of a trial participation decision aid is not yet clear as there has been little assessment to date as to whether the users of these aids—potential (and experienced) trial participants—consider these outcomes to be meaningful. Further input from these stakeholders is required to identify what outcomes they consider should best be used to determine whether these decision support interventions are “effective”. The formal effectiveness evaluation of our decision aid has yet to be conducted, but at this time our anticipated primary outcome is decisional conflict (i.e., how certain someone is of their decision to participate in the trial or not). However, this may be amended in the light of work which is under way (led by our group) to explore stakeholders’ perceptions and build consensus on what outcomes should be considered the core for the evaluation of interventions to improve informed consent to trials [35]; this will inform future evaluations in this space. Also worth considering are longer-term outcomes of trial participation decision aids such as trial retention and future research participation. These trial related outcomes coudl be considered as proxies for decision quality but would have more direct relevance to trial delivery.

c. *Role of process evaluations*

Process evaluations may also be helpful to ensure that trial participation decision aids are being delivered as intended (in other words, to assess fidelity of the intervention in practice). Evidence from a meta-analysis of how clinicians use decision aids in practice highlighted that fidelity to usage instructions of treatment decision aids (in randomised comparisons) was suboptimal and that, in effect, the benefit observed may be greater when used as intended [36]. Process evaluations can also provide important insights for implementation in practice. For example, the study by Politi et al. [15] measured three “implementation outcomes” in their evaluation, namely “time spent on the website, number of visits to the website, and number of participants who visited each page of the website in the [decision aid] group”. Any wider unanticipated impacts on the participant or the wider healthcare delivery system induced by the introduction of the decision aid can also be captured through a process evaluation.

*d. Reporting of the evaluation*

It is important that the results of any evaluation be reported in a detailed and informed manner. Several published reporting guidelines are likely to be relevant and should be considered. These include the Consolidated Standards of Reporting Trials (CONSORT) statement for the reporting of any randomised evaluation [37] or the cluster [38] or stepped-wedge [39] CONSORT extensions as appropriate. The use of the TIDIER (template for intervention description and replication) guidance to ensure adequate reporting of the decision-aid intervention will also be key to allow appropriate replication of the decision aid and to include key aspects of delivery with regard to “who” delivered the decision aid [40]. As decision aids are usually evaluated within a host trial, the reporting guidance for embedded trials should also be considered [41].

### Implementation

One of the biggest challenges for treatment and screening decision aids to date has been mobilisation of findings from evidence into practice and wide scale-up and adoption of these effective interventions into routine care [42]. A range of factors have been put forward as barriers to their adoption in practice, and many focus on changes required at a structural, organisational and individual level [42]. However, the routes to implementation of trial participation decision aids (should further studies evidence benefits) may be less problematic given that many of the structural (legislative and regulatory requirements for informed consent to trials) and organisational (research nurses and others with a dedicated role to seek consent for trials and trial teams with dedicated roles to generate participant information) pathways are already in place. However, for full scale-up, there are important considerations in relation to training those tasked with delivering the trial participation decision aids and ensuring that individuals are not left alone to decide but rather are guided and coached through the decision-making process. Widespread adoption will also require that policy makers recognise the potential added value that these interventions bring to informed choices about trial participation compared with the status quo. As such, the development of a formal plan for implementation and scale-up must also be considered during the design and evaluation of trial participation decision aids.

However, given the context in which decision aids to support trial participation are set (i.e., a live time-limited RCT), both evaluation and implementation must be time-sensitive. Treatment and screening decision aids, once developed, can be effectively used in perpetuity but this is not the case for a trial decision aid. Each trial decision aid is bespoke and has only the lifetime of its host trial. As such, implementation and probably evaluation need to be conceived as much more rapid-cycle than normal. The decision-aid work needs to be finished in the feasibility phase to make it fit for purpose rather than an activity bigger than the trial itself. Whilst there may be enthusiasm from trial teams to develop and implement these tools for trials (unpublished data), ensuring that the development of trial participation decision aids is achieved in a resource-efficient manner (in terms of time, money and expertise) will be key to their success.

### A proposed integrated framework for the development and evaluation of trial participation decision aids

**Fig. 3:**
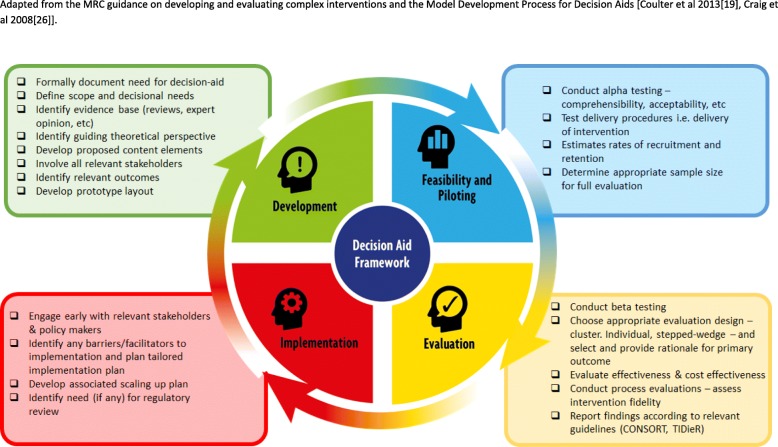
Development and evaluation process for decision aids for people considering trial participation. Adapted from the Medical Research Council (MRC) guidance on developing and evaluating complex interventions and the Model Development Process for Decision Aids (Coulter et al. 2013 [19], Craig et al. 2008 [25]).

Whilst frameworks do exist to help with the development of decision aids for use in clinical treatment decisions, there are no parallel frameworks for the development of decision aids for use in the context of research participation and clinical trial participation. Also, little attention has previously been given to evaluation considerations (and none has acknowledged the cross-learning to decision aids from the complex intervention evaluation literature). We have therefore developed a new and expanded framework to help inform the development and evaluation of trial participation decision aids (Fig. 3). This integrates insights from the MRC complex intervention framework (which we have used as the foundation of this augmented framework), theoretical insights from the ODSF, perspectives integrated from the wider decision-aid literature (including the model development process for treatment decision aids presented by Coulter et al. in 2013 [19]), the complex intervention evaluation literature, and the experiential learning from our empirical testing process. Our proposed model is presented in Fig. 3.

## Conclusions

Building on insights from the decision-aid literature, the evaluation literature and empirical testing, we have developed a new and augmented framework that provides much-needed guidance on the considerations required to develop and evaluate decision aids for clinical trial participation decisions in a robust fashion. Whilst presenting considerations as a worked example, the proposed framework can be used as a systematic and rigorous development and evaluation process for trial participation decision aids which aligns with current guidance on both complex intervention and decision-aid development.

## Data Availability

Not applicable.

## Abbreviations

CONSORT: Consolidated Standards of Reporting Trials
IPDAS: International Patient Decision Aid Standards
MRC: Medical Research Council
ODSF: Ottawa Decision Support Framework
PIL: Patient information leaflet
RCT: Randomised controlled trial
